# Double burden of living alone and in poor socioeconomic conditions among urban-dwelling older Nigerians: a multilevel analysis

**DOI:** 10.1186/s12877-026-07942-5

**Published:** 2026-07-08

**Authors:** Jacob Wale Mobolaji

**Affiliations:** Department of Demography and Social Statistics, Obafemi Awolowo University, Ile-Ife, Nigeria

**Keywords:** Living alone, Loneliness, Social isolation, Double burden, Socioeconomic poverty, Multilevel analysis

## Abstract

Living alone is associated with loneliness, depression and cardiovascular diseases. Living alone and in poor socioeconomic conditions (LAPSC) poses a greater risk of psychosocial health challenges, morbidity and mortality for older persons. The burden of this dyad on population health will likely increase in Nigeria, given the rising migration trends, changing family settings and the country’s ongoing economic crisis affecting household living arrangements and living standards. This study investigates the prevalence and determinants of LAPSC among urban-dwelling older Nigerians. Exploring these is relevant to achieving the sustainable development goals of eradicating poverty (SDG 1) and promoting a healthy life (SDG 3). The study analysed 5,106 weighted samples of older persons aged ≥ 60 from the 2024 Nigeria Demographic and Health Survey. Associations were examined using the multilevel multinomial logistic regression model. Based on the results, 11.0% live alone, of whom 62.6% live in poor conditions. As indicated by the adjusted relative risk ratio (aRRR), the risk of LAPSC was significantly higher for widows (aRRR = 8.12; *p* < 0.001; 95% C.I = 5.81–11.36) than for the married, and in the southern regions as well as other northern regions compared with the North West. However, the risk was 53% lower (aRRR = 0.47; *p* < 0.001; 95% C.I = 0.34–0.65) for women than men; 47% lower for those aged 80 + years than < 70 years; and 46–72% lower for those with primary/secondary (aRRR = 0.54; *p* < 0.001; 95% C.I = 0.39–0.75) and tertiary education (aRRR = 0.28; *p* < 0.001; 95% C.I = 0.17–0.45) compared with those with no formal education. At the community level, communities with middle or high levels of education and communities with low poverty levels had about 56–79% lower risk of LAPSC. The individual- and community-level factors accounted for 11.4% and 43.3% of the variations in LAPSC, respectively. This study suggests individual- and community-level interventions targeting poverty alleviation, access to education, and social inclusion among vulnerable individuals and communities.

## Introduction

Living alone is a major public health concern due to its association with loneliness [1], depression [2], poor psychosocial health [3] and cardiovascular diseases in later life [4]. The trend of living alone is rising globally, [5] reflecting the dynamics of migration, urbanisation, and modernism characterised by shifts in societal structures, economic changes, and individual preferences. Although the proportion of older people living alone in high-income regions (e.g. 27% in the USA and 28% in Europe) surpasses that of low- and middle-income countries (LMIC) (e.g. 9% in sub-Saharan Africa and 11% in Asia-Pacific) [6], the associated burden is of serious concern in LMIC settings with limited resources for older people to live independently. The combination of this living arrangement with material deprivation intensifies vulnerability to negative health consequences, including increased mortality risk, depression, functional decline, hospitalisation and healthcare spending [7].

In developing countries like Nigeria, where formal long-term care systems are limited and ageing-in-place is common, the absence of household co-residents can severely compromise access to care and basic services. Additionally, poverty and poor socioeconomic conditions create substantial barriers to accessing healthcare resources and maintaining adequate nutrition and living conditions [8].

In urban settings of LMIC, older persons living alone and in poor socioeconomic conditions (LAPSC) are exposed to a “double burden” of this dyad, which is further compounded by the challenges associated with urbanisation, including high crime rates, environmental hazards, poverty, high living costs and socioeconomic inequalities [9–12]. Thus, living alone in urban settings exposes older individuals to not only the aforementioned health risks but also to self-harm, falls and domestic abuse.

The living arrangements of older adults have profound implications for their health and social well-being. Those living alone often face diminished social support systems and reduced access to both instrumental and emotional assistance during periods of illness or functional decline [13].

Community characteristics are important factors in studying older persons’ living conditions [14]. However, these have been under-researched in Nigeria. The choices and circumstances that lead older persons to live alone and in poor socioeconomic conditions are often intertwined with the environments in which they reside. Investigating the community-level factors influencing the living conditions of older persons serves as a lens through which community health, economic, and social dynamics can be harnessed to improve well-being in later life. It allows identification of communities where older individuals are at a higher risk of poor health outcomes and provides valuable insights into how their needs can be met.

As the Nigerian population increases, urban areas are characterised by an increasing proportion of older adults, a demographic shift that mirrors the urbanisation process in other countries around the world. These older individuals often navigate the complexities of urban life, where social structures and traditional family support systems are undergoing significant changes [15, 16]. As a result, a growing number of older Nigerians are living alone, with the burdens of economic hardship, social isolation and functional limitations associated with advanced age [17–19].

The situation of older Nigerians is marked by acute socioeconomic vulnerabilities rooted in structural inequalities. The Nigerian government approved a national policy in 2021 to improve the welfare of older citizens [20]. However, the policy is fraught with limited evidence and inadequate content, with poor implementation strategies. The existing pension scheme is limited to the formal sector, poorly managed, and grossly inadequate to address the needs of its beneficiaries [21, 22]. These limitations of the pension scheme leave the majority of pensioners vulnerable and unable to meet their daily financial, nutritional and healthcare needs [23]. Individuals not covered by the pension scheme are more vulnerable, especially where family support – the major safety net – is lacking. Amidst the poor socioeconomic conditions, the ageing experience of urban-dwelling older Nigerians is further worsened by age-unfriendly urban policies, [24] and inadequate social amenities. These poor conditions have implications for health outcomes in later life, yet their intersection with living arrangements remains underexplored in Nigeria.

Factors associated with living alone are multifaceted, encompassing demographic attributes such as age, gender, marital status, and childlessness, [25–28] as well as economic considerations, cultural norms, and urbanisation trends [29, 30]. Also, the HIV/AIDS epidemic has contributed to living alone by some older persons who have lost their children [31, 32]. Besides, childlessness, [29] marriage and labour force participation of adult daughters, [33] migration, emphasis on nucleated family, modernization and the changing traditional family setting [34] have also contributed to living alone among older persons. However, the majority of this evidence is in the context of either high-income countries or Asia, with limited evidence from Africa, especially from Nigeria, with social, economic and cultural diversities. Understanding these factors in the Nigerian context is essential not only for social researchers but also for policymakers, as they have far-reaching implications for social support systems.

This study aims to investigate the prevalence and predictors of LAPSC among older persons in urban Nigeria. It delves into the intricate interplay of individual and community factors that contribute to the vulnerability of older individuals living alone and, at the same time, in impoverished conditions. By examining the prevalence and determinants of this dyad, the study aims to provide valuable insights that can inform policy and intervention strategies tailored to the unique needs of older persons. Through rigorous analysis and empirical investigation, this study contributes to a deeper understanding of the challenges faced by older individuals living alone in urban Nigeria and, ultimately, proposes evidence-based recommendations that enhance their quality of life and well-being.

## Methods

### Data sources and sample design

The study analysed the household member recode (PR) file of the 2024 Nigeria Demographic and Health Survey (DHS). The survey was based on a stratified two-stage cluster design involving 1,400 clusters, 701 in urban areas and 699 in rural areas, across the country’s six geopolitical zones. The dataset contains relevant variables to the study, including individual background, socioeconomic characteristics and household living arrangements. The details of the sample design and data collection methods were published in the DHS report [35]. A weighted total sample of 5,106 elderly household members aged ≥ 60 years and their relevant data were extracted and analysed for this study.

### Variable measurement

In this study, the outcome variable – living alone and in poor socioeconomic conditions – is a computation from two variables: living arrangements and socioeconomic conditions. Older persons living alone (coded 1) or living with someone (coded zero) were generated from the household living arrangements. The socioeconomic condition was measured using the United Nations standard of living indicators, including housing conditions, drinking water, toilet facilities, cooking fuel and electricity [36]. Household members are in poor condition in each indicator if they live in houses with mud, thatch, and dung mixtures; drink water from unprotected sources or the distance to drinking water is more than 15 min; lack modern or closed toilet facilities; cook household food with charcoal, shrubs/grass, and wood and the like; and have no electricity.

Respondents were scored based on these indicators; the scores were summed up, and the median score was obtained. Respondents whose scores were greater than or equal to the median score were categorised as living in poor socioeconomic conditions (coded 1), while the rest were categorised otherwise (code = 0). Combining the two variables, the outcome variable comprises three different scores: 0, 1 and 2, indicating three categories: Living alone and in poor socioeconomic condition (LAPSC) (score = 2); either living alone or in poor condition without experiencing both (score = 1); neither alone nor poor (score = 0).

The independent variables are the respondents’ characteristics (age, sex, marital status and level of education) and community characteristics, including the region of residence, community-level education and poverty. Clusters were used as the geographic units to define a community in this study. Overall, 697 urban clusters with an average cluster size of 115 (Standard Deviation = 38.8) were used to compute the community-level variables. The number of clusters and cluster size are adequate for assessing the distribution of educational and poverty levels of the communities in which older persons reside [37–39]. The community-level education was computed based on the proportion of household members with various levels of education in the community. The proportions were divided into tertiles (low, middle and high). The same procedure was used for computing the community-level poverty using the wealth quintile. Based on the proportion of households in the poorest, poorer, middle, richer and richest wealth quintiles in each community, the poverty levels of the community were divided into tertiles: high (indicating communities with a high proportion of households in the poorer and poorest wealth quintiles), middle and low (communities with the lowest proportion of households in the poorer and poorest wealth quintiles). Tertiles computed from the community variables were weighted using probability weights to reflect the true educational and poverty distributions in the population.

### Data analysis

The data were analysed at the univariate level using frequency and percentage distributions. For the multivariable analysis, a multilevel multinomial logistic regression model was employed to examine the individual- and community-level factors associated with LAPSC. This analytical approach accounts for the hierarchical structure of the data, with individuals nested within communities, and allows estimation of relative risk ratios (RRR) for categories of the outcome relative to the reference category (neither alone nor poor). Two models were specified: Model 1 included individual-level variables, and Model 2 additionally adjusted for community-level variables and region of residence. Since this study aims at investigating those who are vulnerable to both LAPSC, interpretations focused mainly on those in the LAPSC category, with minimal attention to those who experience only one of living alone or in poor condition, the second category. All analyses incorporated sample weights to adjust for the complex survey design and were conducted using Stata version 17.0 [40]. Statistical significance was assessed at the 5% level, and 95% confidence intervals (CI) were reported. Variance partition coefficients (VPC) and proportional change in variance (PCV) were calculated to quantify the relative contributions of individual- and community-level factors to variations in LAPSC.

Missing data analysis was conducted to ensure unbiased results. The diagnosis of the missing data indicated no missing values in all the variables included in this study. Thus, all analyses were based on complete data. A multicollinearity test was also conducted to detect high correlations among the independent variables. The Variance Inflation Factor (VIF) result indicates no multicollinearity of concern among the independent variables.

### Ethical considerations

This study adhered to the guidelines of the Helsinki Declaration in its utilisation of the DHS, a program coordinated by ICF International. The survey complied with local legislation, institutional requirements, and the Helsinki Declaration, as informed consent to participate was obtained from all the participants of the study. The DHS protocol was approved by both the National Health Research Ethics Committee of Nigeria (NHREC) and the ICF Institutional Review Board (IRB). The IRB-approved protocol for the DHS public-use datasets ensures no identification of respondents, households, or sample communities. For this study, written approval to access the DHS data was obtained from ICF International.

## Results

Over half of the respondents included in the secondary analysis for this study were males (54.1%), largely below age 70 years (60%), married (65.2%) and had below secondary education (60.2%). About one-third of the respondents were from the South-West (37.0%), with about 16.0% each from North West and South-South. North Central was the least represented (< 10.0%) (Table 1 in Appendix). This regional distribution reflects the proportion of older people in the South-West compared to other regions.

### Proportions of older Nigerians living alone in poor socioeconomic conditions

The results indicate that 11.0% of urban-dwelling older Nigerians live alone, and 58.7% are in poor condition. Of those living alone, 62.6% live in poor socioeconomic conditions. Overall, 37.2% of the respondents neither live alone nor are in poor condition, 51.8% were poor but not living alone, while only 4.1% were living alone but not poor (Table 2 in Appendix). The proportion of older adults living alone and in poor socioeconomic conditions (6.9%), though similar across age groups, was relatively higher among females (8.7%) compared to males (5.3%), with the highest prevalence reported among the single/divorced (22.3%) and widows (14.7%), especially those without formal education, living in communities with low educational and poverty levels, and those in the three southern regions (Table 3 in Appendix).

### Factors associated with living alone in poor socioeconomic conditions

#### Fixed effects

The fixed effect results reveal the associations between various factors (sex, age, marital status, education, community characteristics, and region of residence) and the risk of living alone or in poor condition or doubling both (Table 4 in Appendix). Examining the individual-level variables in Model 1, the fixed-effect results, estimated using the Relative Risk Ratios (RRR), indicate that women had a 54% lower risk of LAPSC (RRR = 0.46; *p* < 0.001; 95% C.I.=0.33–0.64) compared to men. Similarly, the risk was lower by 45% among those aged 80+ (RRR = 0.55; *p* < 0.001; 95% C.I.=0.37–0.82) and by 49–79% among those with primary/secondary education (RRR = 0.51; *p* < 0.001; 95% C.I.=0.38–0.70) or tertiary education (RRR = 0.21; *p* < 0.001; 95% C.I.=0.13–0.34). Conversely, the risk of LAPSC was eight-fold higher among widows (RRR = 8.47; *p* < 0.001; 95% C.I.=6.04–11.87) compared to non-widows. These associations were consistent when adjusted for other factors (Model 2).

At the community level, older persons living in communities with middle (RRR = 0.54; *p* < 0.001; 95% C.I.=0.34–0.85) or high levels of education (RRR = 0.31; *p* < 0.001; 95% C.I.=0.18–0.54) had about 46–69% lower risk of LAPSC compared with their counterparts living in communities with low levels of education. When adjusted for individual factors (Model 2), the association remained significant. Those who live in communities with low poverty levels also had a significantly lower risk of doubling the two living conditions compared with those who live in communities with high/middle poverty levels; the association was consistent when adjusted for individual factors in Model 2. Older adults in other regions had multiple-fold higher risk of LAPSC compared to their counterparts in the North-West region (Model 1), with the highest risks in the South-West (RRR = 13.82; *p* < 0.001; 95% C.I.=6.45–29.60), South-South (RRR = 10.96; *p* < 0.001; 95% C.I.=4.97–24.17) and South-East (RRR = 7.51; *p* < 0.001; 95% C.I.=3.41–16.55) regions. When adjusted for other factors in Model 2, the associations remained consistent.

The fixed-effect results show that the risk of living alone or in poor condition without doubling both, and that individual as well as community factors also follow similar patterns, comparable to those who experienced both LAPSC. However, in both Models 1 and 2, a significantly higher risk of living alone or in poor condition was observed in other regions, except in the North-Central and South-West (in Model 1), compared with the North-West. The associations with the community’s level of education were also significant, as observed in LAPSC. 

#### Random effects

The random effects of the multilevel model in Table 4 in Appendix, depicted by the variance and variance partition coefficients (VPC), explain the variability in outcomes at the individual and community levels. The VPC in the empty model indicates that 42.7% of the variance in LAPSC is at the community level. The proportional change in variance (PCV) indicates that individual-level factors accounted for 11.4% of the variations in the risk of LAPSC, while community-level factors accounted for 43.3%. For either living alone or living in poor socioeconomic conditions, without experiencing both, the VPC indicates that 23.5% of the variance is at the community level. The PCV indicates that individual-level factors explain 15.2% of the variations in the risk of either living alone or in poor socioeconomic conditions, while community-level factors account for 47.1% of the variations (Table 4 in Appendix).

## Discussion

Nigeria accounts for a substantial proportion of Africa’s elderly population and faces rapid demographic ageing, yet comprehensive epidemiological research on older persons’ welfare remains limited. This study provides population-level data on living arrangements and socioeconomic security among Nigerian older adults. It aims to examine the double vulnerabilities of LAPSC and the associated individual as well as community factors accounting for the vulnerabilities. The study’s conceptualisation of LAPSC as a combined outcome acknowledges that living alone and poverty may not necessarily be independent risks but co-occurring vulnerabilities, a framing that better reflects the complexity of older people’s marginalisation in African contexts with low socioeconomic conditions and weak formal social safety nets. The findings from this study provide a policy guide for addressing the needs of vulnerable older populations in Nigeria.

The prevalence of LAPSC among older Nigerians sheds light on a critical aspect of the country’s ageing population. The findings reveal important insights into the challenges faced by these Nigerians, particularly in urban areas. The 11.0% prevalence of LAPSC indicates a level of social isolation among older individuals, which has adverse effects on their mental and emotional well-being. The finding aligns broadly with patterns observed in other developing regions [41], though with varied magnitudes. The finding that 62.6% of those living alone experience poor socioeconomic conditions in this study is notably high, reflecting a double burden of isolation and severe socioeconomic vulnerabilities that significantly impact their quality of life. This elevated proportion suggests a higher concentration of poverty among the lone-dwelling older persons in Nigeria compared to some Asian contexts, like Thailand, with a lower proportion (39%) of older adults living alone and below the national poverty line [42]. This disparity possibly reflects differential access to pension systems, family support networks, and formal social protection mechanisms in sub-Saharan Africa.

The gender and regional variations in the proportion of older persons living alone and in poor socioeconomic conditions reveal a higher prevalence among females (8.7%) than males (5.3%), and in southern regions (7.3–9.2%) compared to the North, especially the North-West (2.0%). The gender disparity is consistent with evidence from other contexts [43–45]. This, in part, may be due to a higher concentration of widowhood among older women, with limited economic power and financial autonomy, who have probably lost their economic power after their husband’s death. The regional disparities also suggest a regional dimension to the challenge, indicating that a higher concentration of older individuals living alone and in poor conditions in certain regions requires region-based interventions.

This study makes a unique contribution to understanding the vulnerabilities of older persons in an LMIC context like Nigeria. The multilevel design examining individual characteristics, community factors, and regional influences simultaneously in a large, nationally representative sample is relatively uncommon in African gerontological research. Most research on ageing in sub-Saharan Africa has focused narrowly on either individual risk factors or has been qualitative in nature [1, 15, 16, 18, 46, 47] The fixed-effects results of the multilevel analysis revealed that some individual factors, particularly widowhood, were associated with increased risk of LAPSC. This association is also reflected in the prevalence of LAPSC by marital status, with single/divorced persons (22.3%) and widows (14.7%) showing higher prevalence compared to married ones (2.2%). This finding aligns with evidence from China, India, and Europe, [48–50} indicating that marital dissolution, particularly through spousal death, substantially increases vulnerability to both living alone and economic hardship.

Age was inversely related to LAPSC risk, with adults aged 80 + having 45% lower risk compared with those aged < 70 years old. This relationship agrees with existing evidence suggesting that the risk of living alone decreases with age [25]. This pattern may reflect the likelihood that the oldest-olds are functionally limited and no longer able to live alone. Thus, such individuals may benefit from increased family support based on old-age considerations, unlike younger individuals who are assumed to be functionally and economically active. It may also reflect survival bias, where the most economically vulnerable elderly may not survive to advanced age due to other health system deficiencies. However, the finding contrasts with a few studies suggesting either a heightened vulnerability, [51] or no significant effect [47] among the oldest-old populations. These disparities may be linked to contextual and methodological variations.

Similarly, a higher level of education was associated with a lower risk of LAPSC. The higher prevalence among individuals with no formal education (8.9%) compared to those with tertiary education (3.9%) also supports this association. This finding may be linked to the role of education in enabling access to good employment opportunities, higher income, and the ability to afford a paid caregiver or caregiving home. Similar associations between education and socioeconomic conditions have been reported in other populations [52, 53]. This consistency suggests that the role of education in promoting economic security and health literacy operates across socioeconomic contexts, though the magnitude of protection may vary.

The VPC of the random effect results in the empty model indicates that 42.7% of the variance in LAPSC is at the community level. This quantification provides empirical evidence that place-based interventions may be especially important for this population. While the PCV indicates that individual-level factors account for only 11% of the variation in older persons’ LAPSC experience, 43.3% of the variation is attributable to community-level factors examined, indicating a significant finding and policy implications. Older persons in communities with middle or high educational levels had about 46–69% lower risk of LAPSC compared with those in low-education communities. Community poverty level also follows similar patterns. These community effects emphasise how neighbourhood social infrastructure shapes individual outcomes. These findings agree with a Nepalese study, which demonstrated that neighbourhood-level social support infrastructure was associated with a major depressive disorder incidence among adults in high-poverty areas [54]. This suggests that similar mechanisms may operate for elderly living arrangements and poverty outcomes.

The regional variations, with South-West, South-South, and South-East regions showing about 7-fold to 13-fold higher risk of LAPSC compared to the North-West, reflect variations in their sociocultural and socioeconomic indicators. This regional disparity may be linked to a lower proportion of older adults living alone in the North, where African family constellations are relatively more preserved compared to the southern regions, with sociocultural transformations resulting from modernisation, migration patterns, and socioeconomic developments.

### Strengths and limitations of the study

This study demonstrates some methodological strengths. The large and representative sample of urban-dwelling older persons in this study provides robust estimates with adequate statistical power to detect associations even among subgroups. The multilevel analytic approach appropriately addresses the hierarchical data structure, with individuals nested within communities and regions, enabling estimation of contextual effects independent of individual characteristics. This approach provides a nuanced understanding of how context shapes an individual’s vulnerability profiles. Also, focusing on urban areas provides important data for urban policy while potentially identifying vulnerable urban-dwelling elderly who may receive less programmatic attention than their rural counterparts.

A few limitations are noteworthy, however. The study is a cross-sectional design in which both the outcome and exposure variables are measured simultaneously. Hence, associations found in this study do not imply causal inferences. In addition, since the study was based on secondary data, explanatory variables included in the model were limited to those available in the dataset. Also, restrictions to urban areas limit generalizability to rural dwellers, among whom living arrangements, poverty patterns, and access to support systems may differ substantially from those of urban dwellers. The rural-urban gradient in poverty and social support means findings cannot be directly extrapolated nationally. Future research should examine rural patterns and urban-rural comparisons. In addition, poverty measurement was based on asset indices of standard-of-living indicators. The indices provide proxy measures of poverty, though they may not fully capture income adequacy, subjective poverty, or vulnerability to economic shocks. Finally, the study does not distinguish between different levels of poor socioeconomic conditions, treating poverty as dichotomous. Ordinal or continuous poverty measures could provide a more granular understanding of severity gradients and enable identification of moderately versus severely poor older persons. However, these limitations do not affect the validity of the findings of this study.

## Conclusion

This study estimated the proportion of older Nigerians living alone and in poor socioeconomic conditions, with notable sociodemographic disparities. The findings demonstrate the lower risk associated with a higher level of education, and marked risks associated with widowhood, marital dissolution and region of residence. These patterns reflect Nigeria’s distinctive cultural, economic, and epidemiological contexts. The finding that community-level factors account for approximately three-quarters of the variance in LAPSC underscores that elderly vulnerability in the African context, especially in Nigeria, cannot be adequately addressed through individual-focused interventions alone. Policy responses must simultaneously address disparities through targeted community programs.

### Policy implications

This study suggests prioritisation of community-level interventions alongside individual-focused approaches. Given that community factors substantially shape LAPSC outcomes, policies focusing exclusively on individual characteristics risk limited effectiveness. Community-based programmes should target poverty alleviation, access to education, and social cohesion in vulnerable neighbourhoods. In addition to community-based programmes, interventions targeting vulnerable individuals, like widow support programmes combining financial assistance, social connectivity enhancement, and potentially housing support, could benefit vulnerable widows and widowers. The high prevalence of poverty among lone-living older persons suggests current contributory pension system, covering only a minority of older Nigerians, is inadequate. Expanded non-contributory pensions, particularly for older persons lacking formal employment history, would reduce poverty while potentially enabling continued independent living. Summarily, this study contributes essential epidemiological evidence for older persons policy efforts in Nigeria and demonstrates that addressing the well-being of older Nigerians requires integrated individual-community policy approaches grounded in locally relevant evidence on older people’s vulnerability patterns.

## Data Availability

The dataset analysed in the current study is available in the Demographic and Health Survey (DHS) program repository: [ https://dhsprogram.com/data/available-datasets.cfm ].

## Appendix

**Table 1:** Background characteristics of the respondents

	Frequency (*N* = 5106)	%
Sex		
Male	2,764	54.1
Female	2,342	45.9
Age		
< 70	3,066	60.0
70–79	1,443	28.3
80+	597	11.7
Marital status		
Married	3,331	65.2
Widowed	1,572	30.8
Single/divorced	203	4.0
Highest level of education		
No formal education	1,795	35.2
Primary	1,276	25.0
Secondary	1,006	19.7
Tertiary	1,029	20.2
Region		
North West	812	15.9
North East	618	12.1
North Central	424	8.3
South East	555	10.9
South South	807	15.8
South West	1,891	37.0

**Table 2:** Crosstabulation of older persons living alone and in poor conditions

	Living condition		
	Not in poor socioeconomic conditions (*n* = 2,110)	In poor socioeconomic conditions (*n* = 2,996)	Total (*N* = 5,106)
Living arrangement			
Not living alone	1,901 (41.8)	2,645 (58.2)	4,546 (89.0 ^c^)
Living alone	209 (37.4)	351 (62.6)	561 (11.0 ^c^)
Total	2,110 (41.3)	2,996 (58.7)	5,106 (100.0)

c - column percentages

**Table 3:** Univariate association between LAPSC and sociodemographic characteristics and community-level variables

Sociodemographic characteristics	Living arrangements and conditions			Total	χ^2^
	Neither living alone nor in poor condition *n* (%)	Living alone / in poor condition *n* (%)	LAPSC *n* (%)		
Overall	1,901 (37.2)	2,854 (55.9)	351 (6.9)	5,106	
Sex					
Male	1076 (38.9)	1541 (55.8)	147 (5.3)	2764	13.125***
Female	825 (35.2)	1313 (56.1)	205 (8.7)	2342	
Age					
< 70	1186 (38.7)	1685 (54.9)	195 (6.4)	3066	1.646
70–79	512 (35.4)	819 (56.8)	112 (7.8)	1443	
80+	203 (34.0)	351 (58.7)	43 (7.3)	597	
Marital status					
Married	1399 (42.0)	1857 (55.8)	75 (2.2)	3331	62.864***
Widowed	450 (28.6)	891 (56.7)	231 (14.7)	1572	
Single/divorced	52 (25.5)	106 (52.2)	45 (22.3)	203	
Highest level of education					
No formal education	411 (22.9)	1224 (68.2)	160 (8.9)	1795	50.709***
Primary/Secondary	899 (39.4)	1233 (54.0)	150 (6.6)	2282	
Tertiary	591 (57.4)	398 (38.6)	40 (3.9)	1029	
Community-level variables					
Community Education					
Low	387 (22.6)	1205 (70.3)	122 (7.1)	1714	27.985***
Middle	636 (37.6)	921 (54.4)	136 (8.0)	1693	
High	878 (51.6)	728 (42.9)	93 (5.5)	1699	
Community poverty					
Low	273 (16.0)	1292 (75.8)	141 (8.2)	1705	54.836***
Middle	711 (41.4)	875 (51.1)	129 (7.5)	1714	
High	917 (54.4)	688 (40.8)	82 (4.8)	1686	
Region					
North West	276 (34.0)	520 (64.0)	16 (2.0)	812	8.065***
North East	122 (19.7)	456 (73.8)	40 (6.5)	618	
North Central ^ref^	174 (41.0)	229 (53.9)	22 (5.1)	424	
South East	211 (38.0)	303 (54.7)	41 (7.4)	555	
South South	273 (33.8)	475 (58.9)	59 (7.3)	807	
South West	845 (44.7)	872 (46.1)	173 (9.2)	1891	

*** *p* < 0.001; χ^2^ Chi-Squared; LAPSC living alone and in poor socioeconomic conditions

**Table 4:** Multilevel multinomial logistic regression models of individual and community factors associated with LAPSC in older Nigerians

	Empty model		Model 1		Model 2	
	Living alone / in poor condition	LAPSC	Living alone/ in poor condition	LAPSC	Living alone/ in poor condition	LAPSC
			RRR (95% C.I.)	RRR (95% C.I.)	aRRR (95% C.I.)	aRRR (95% C.I.)
Level 1 (individual level)						
Sex						
Male ^ref^			1.00	1.00	1.00	1.00
Female			0.73 (0.62–0.86)***	0.46 (0.33–0.64)***	0.78 (0.66–0.92)**	0.47 (0.34–0.65)***
Age						
< 70 ^ref^			1.00	1.00	1.00	1.00
70–79			0.88 (0.75–1.04)	0.86 (0.65–1.15)	0.90 (0.76–1.06)	0.84 (0.63–1.13)
80+			0.70 (0.55–0.89)**	0.55 (0.37–0.82)**	0.71 (0.56–0.90)**	0.53 (0.35–0.79)**
Marital status						
Non-widow ^ref^			1.00	1.00	1.00	1.00
Widows			1.44 (1.19–1.75)***	8.47 (6.04–11.87)***	1.45 (1.20–1.76)***	8.12 (5.81–11.36)***
Highest level of education						
No formal education ^ref^			1.00	1.00	1.00	1.00
Primary/Secondary			0.49 (0.41–0.60)***	0.51 (0.38–0.70)***	0.59 (0.49–0.72)***	0.54 (0.39–0.75)***
Tertiary			0.25 (0.19–0.31)***	0.21 (0.13–0.34)***	0.34 (0.26–0.43)***	0.28 (0.17–0.45)***
Level 2 (community level)						
Community Education						
Low ^ref^			1.00	1.00	1.00	1.00
Middle			0.55 (0.39–0.77)***	0.54 (0.34–0.85)**	0.60 (0.43–0.84)**	0.61 (0.38–0.98)*
High			0.40 (0.27–0.58)***	0.31 (0.18–0.54)***	0.53 (0.36–0.78)**	0.44 (0.25–0.79)**
Community poverty						
High/Middle ^ref^			1.00	1.00	1.00	1.00
Low			0.29 (0.22–0.40)***	0.19 (0.13–0.30)***	0.31 (0.23–0.42)***	0.21 (0.14–0.32)***
Region						
North West ^ref^			1.00	1.00	1.00	1.00
North East			1.78 (1.07–2.94)*	5.11 (2.19–11.93)***	1.68 (1.01–2.80)*	4.39 (1.85–10.44)**
North Central			1.05 (0.66–1.68)	3.29 (1.41–7.69)**	1.14 (0.71–1.83)	3.42 (1.44–8.12)**
South East			1.73 (1.10–2.72)*	7.51 (3.41–16.55)***	1.90 (1.20–3.02)**	6.50 (2.89–14.60)***
South South			2.38 (1.53–3.70)***	10.96 (4.97–24.17)***	2.70 (1.72–4.23)***	10.15 (4.51–22.84)***
South West			1.49 (0.98–2.27)ϯ	13.82 (6.45–29.60)***	1.61 (1.05–2.47)*	12.87 (5.91–28.03)***
Random Effects						
Level 1 (individual-level)						
Variance (SE)	1.91	2.45	1.62	2.17	1.12	1.44
VPC	36.7	42.7	33.0	39.7	25.4	30.4
PCV (%)	ref	ref	15.2	11.4	41.4	41.2
Level 2 (community-level)						
Variance (SE)			1.01	1.39		
VPC			23.5	29.7		
PCV (%)	ref	ref	47.1	43.3		

*Abbreviations*: *SE *standard error, *VPC *variance partition coefficient, *PCV *proportional change in variance, *C.I*. confidence interval, *LAPSC *living alone and in poor socioeconomic conditions, *RRR *relative risk ratio, *aRRR *adjusted relative risk ratio, ref reference category, Empty model no independent variable in the model, Model 1: includes all individual variables and all community variables separately, Model 2: includes all individual and community variables together in the model

* *p* < 0.05; ** *p* < 0.01; *** *p* < 0.001

## Abbreviations

LAPSC: Living Alone and in Poor Socioeconomic Conditions
LMIC: Low- and Middle-Income Countries
SDG: Sustainable Development Goals
SSD: Single, Separated or Divorced
VPC: Variance Partition Coefficients
PCV: Proportional Change in Variance
DHS: Demographic and Health Survey
RRR: Relative Risk Ratio
C.I.: Confidence Interval
